# Lab-on-Chip Platform for Culturing and Dynamic Evaluation of Cells Development

**DOI:** 10.3390/mi11020196

**Published:** 2020-02-14

**Authors:** Agnieszka Podwin, Danylo Lizanets, Dawid Przystupski, Wojciech Kubicki, Patrycja Śniadek, Julita Kulbacka, Artur Wymysłowski, Rafał Walczak, Jan A. Dziuban

**Affiliations:** 1 Faculty of Microsystem Electronics and Photonics, Wrocław University of Science and Technology, 50-370 Wrocław, Poland; danylo.lizanets@pwr.edu.pl (D.L.); wojciech.kubicki@pwr.edu.pl (W.K.); patrycja.sniadek@pwr.edu.pl (P.Ś.); artur.wymyslowski@pwr.edu.pl (A.W.); rafal.walczak@pwr.edu.pl (R.W.); jan.dziuban@pwr.edu.pl (J.A.D.); 2 Department of Molecular and Cellular Biology, Faculty of Pharmacy, Wrocław Medical University, 50-367 Wrocław, Poland; dawid.przystupski@gmail.com (D.P.); julita.kulbacka@umed.wroc.pl (J.K.)

**Keywords:** lab-on-chip platform, cell culture, cancer research, IVM (In Vitro Maturation), taxis effects, behavioral analysis

## Abstract

This paper presents a full-featured microfluidic platform ensuring long-term culturing and behavioral analysis of the radically different biological micro-objects. The platform uses all-glass lab-chips and MEMS-based components providing dedicated micro-aquatic habitats for the cells, as well as their intentional disturbances on-chip. Specially developed software was implemented to characterize the micro-objects metrologically in terms of population growth and cells’ size, shape, or migration activity. To date, the platform has been successfully applied for the culturing of freshwater microorganisms, fungi, cancer cells, and animal oocytes, showing their notable population growth, high mobility, and taxis mechanisms. For instance, circa 100% expansion of porcine oocytes cells, as well as nearly five-fold increase in *E. gracilis* population, has been achieved. These results are a good base to conduct further research on the platform versatile applications.

## 1. Introduction

The idea of miniaturization has become a major tendency in a wide range of science, especially covering biomedical fields, where fast, reliable, and portable instrumentation is essential. In the last two decades, special attention has been paid towards the development of new tools dedicated to basic laboratory standards, such as cell culture [1,2,3].

Typically, the process of cell culturing is conducted on flat substrates, for example, Petri dishes or culture bottles. As the cells in their natural environment are constantly subjected to dynamic, various influences, this model do not imitate in vivo conditions accurately. According to other papers [4,5], cultures established in Petri dishes may be characterized by notable degradation of cells, changes in their metabolism, or gene expression.

With regard to other works [6,7,8], one of the most popular ways to minimize the risk of inappropriate cells’ development is to provide constant media flow in the culture area, mimicking slightly an in vivo perfusion system. As the macroscale tools, for example, Petri dishes, are dedicated basically to stationary cultures, the solution of precise and repeatable media dosage to the cells falls here exactly within the scope of microfluidic lab-on-chip (LOC) instrumentation [9,10].

The achievement of reliable and effective cell cultures on-chip recently constitutes one of the most popular research trends, allowing for further, more sophisticated cell studies, for example, drug resistivity, electroporation, taxis, and quality assessment [11,12,13,14,15,16,17]. The literature on the subject presents many examples of cell culture LOCs, but their real use in biomedical units is still quite limited. On the basis of the state-of-the-art analysis, one of the reasons for this issue may be the uncertain biocompatibility of LOCs, from which construction materials usually differ, used typically for macroscale culturing tools—glass or polystyrene [18,19]. The dominant material here is polydimethylosiloxane (PDMS), which, unfortunately, might be considered as not fully bioinert, preventing the achievement of reliable and long-term cell culturing [18,20,21,22].

In order to improve PDMS biocompatibility, a visible tendency to use special layers, for example, Parylene, Kraton, or hydrogel-based layers, has been observed [23,24,25,26]. Nevertheless, these solutions are still rather PoC (Proof of Concept), requiring advanced technology and dependable statistics to achieve fully-featured cell culturing platforms.

Biocompatibility of medical instrumentation is not the only issue that influences the development of cell culturing platforms. Another problem here is a fabrication of microfluidic devices that are typically dedicated to perform single, well-defined experiments. There is a lack of multi-tasking platforms in which it would be possible to culture and provide behavioral analysis of the biological objects in response to stimulation with different external factors, for example, chemical, thermal, optical, and electrical.

**Figure 1:**
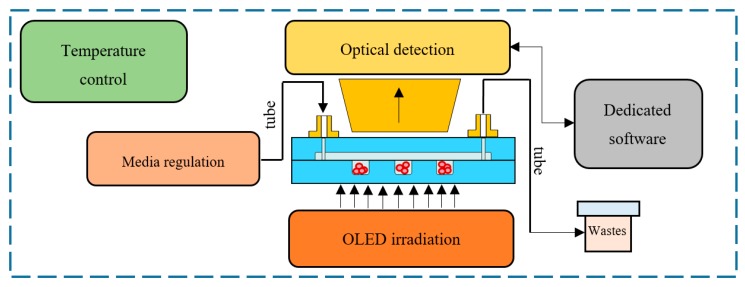
A schematic diagram of the lab-on-chip (LOC) platform.

As the matter of cell culturing LOCs is still broadly investigated in the literature, this article presents a concept of the microfluidic platform, ensuring culturing and evaluation of biological potential of different biological objects in a versatile and fully biocompatible way. A family of all-glass, reusable lab-chips has been fabricated here to provide culturing of freshwater micro-organisms, mushroom representatives, human cancer cells, and animal oocytes. All the necessary equipment allowing for simultaneous investigation of the cells in response to chemical, optical, and thermal stimulation has been also applied. Eventually, dynamic, software-enhanced metrological characterization of the cultures could be done to unambiguously assess the development potential of the cultured objects within a single microfluidic device. The scheme of the platform is shown in Figure 1.

## 2. Materials and Methods

### 2.1. Elements of the LOC Platform

**Figure 2:**
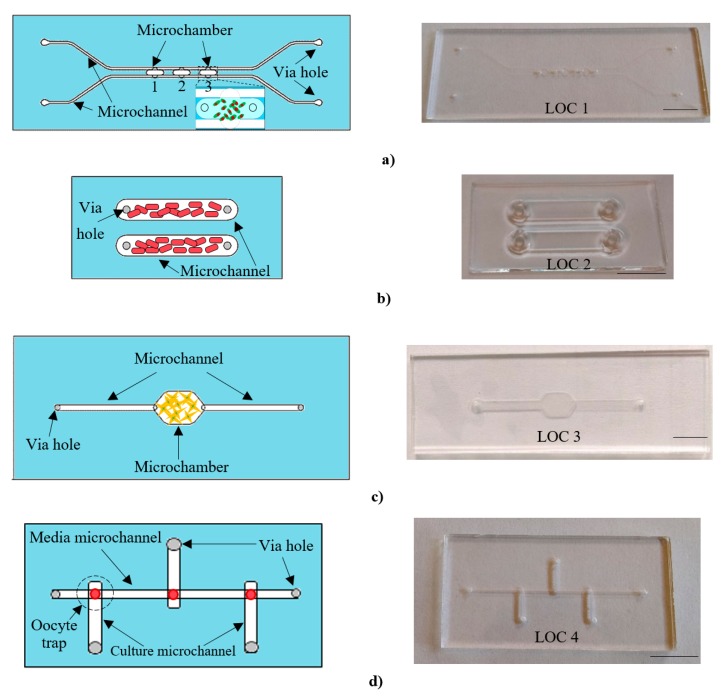
A family of all-glass lab-chips dedicated to cell culturing of various biological objects: (**a**) freshwater micro-organisms—*Euglena gracilis* (volume of the single microchamber: ~1 μL, surface area: 4 × 2.5 mm^2^), (**b**) mushrooms—*Cladosporium macrocarpum* (volume of the microchannel: ~23.5 μL, surface area: 16.8 × 3.5 mm^2^), (**c**) human cancer cells SKOV-3 (volume of the microchamber: ~8.5 μL, surface area: 9.5 × 6 mm^2^), (**d**) porcine oocytes (volume of the culture microchannel: ~0.6 μL, surface area: 7 × 0.5 mm^2^). Scale bar—1 cm.

All-glass lab-chips were fabricated utilizing commercially available borosilicate glass slides (Borofloat 3.3, Schott, Mainz, Germany). In the literature on the subject, a variety of techniques ensuring fabrication of microstructures in glass substrates can be distinguished, for example, laser ablation [27,28] or sandblasting; nevertheless, in this study, typical micromachining processes including xurography, wet chemical etching, mechanical drilling of via holes, and thermal bonding were applied to achieve final LOC structures (Figure 2). The pattern of the microchannels/microchambers was formed by submerging the glass slides in a solution of 50% HF/69% HNO_3_ (10:1 v/v), etching speed: ~3 μm/min. Next, via holes were drilled to provide the entrance for the biological samples. After cleaning the substrates with trichloroethylene, acetone, IPA, deionized water, and Piranha solution (H_2_SO_4_:H_2_O_2_, 3:1, v/v), they were bonded in a furnace at a temperature of 80 °C (Figure 2a) or 650 °C (Figure 2b–d).

The geometry of the chips was dictated by the features of the studied objects, differing notably in size, shape, and migration activity. Depending on the type of the cultures, the optimal construction of the chip was proposed (Table 1), encompassing, for instance, internal trapping structures (porcine oocytes maturation) or two bypass microchannels, in the case of chemotaxis surveys of *E. gracilis* [29].

**Figure 3:**
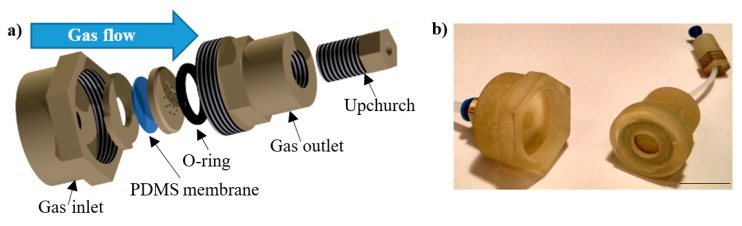
Three-dimensional printed microflow regulator providing gaseous media supply for the cultures on the LOC platform [30]: (**a**) schematic view, (**b**) regulator at a glance. Scale bar—1.5 cm. PDMS, polydimethylosiloxane.

As the culturing on the platform was dedicated to various cells, the platform was equipped with the elements ensuring the delivery of nourishment in both gaseous or liquid form. In the case of the gaseous media, a solution of 3D membrane-based gas regulator was used (Figure 3) [30], providing precise and repeatable flow in the range of 0.1–50 μL/min. With regard to liquid media supply, a commercial solution of MEMS micropump (model: mp6, Bartels Mikrotechnik, Dortmund, Germany) was applied. Standard microfluidic connectors (Nanoport Assembly, Idex H&S, Oak Harbor, WA, USA) were used at the LOC interface to enable repeatable dosing of the culturing media through the ferrule of 1/16′’ diameter (PFA tubing, Idex H&S, Oak Harbor, WA, USA).

**Figure 4:**
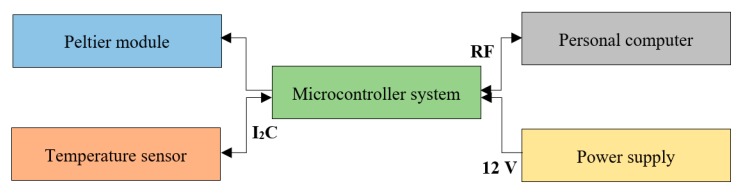
Block diagram presenting temperature module operation.

Another essential part of the microfluidic platform was the temperature module (Figure 4), allowing for the portable and convenient cell culturing, performed outside the standard incubator. The module was designed to control the temperature (ambient temperature—42 °C), especially in the case of the mammalian cells cultures, for example, human cancer cells (SKOV-3) or animal oocytes (porcine).

The operation of the module refers to the initialization of programmable PID (proportional–integral–derivative) algorithm. Digital temperature sensor (model: MCP9800, Microchip Technology, Chandler, AZ, USA) communicates with the microcontroller (MKE02Z64VLD2, NXP Semiconductors, Eindhoven, The Netherlands), which, based on its indication, steers the Peltier module (model: TEC1-07108 Stonecold, Transfer Multisort Elektronik TME, Łódź, Poland) in a closed feedback loop to maintain the desired temperature on-chip. The temperature module communicates with the PC wirelessly by the RF device (model: nRF24L01+, Botland, Gola, Poland), Figure 4. Observable temperature fluctuations do not exceed ±0.25 °C here.

As the source of cells irradiation, for the first time, the OLED display (model: microOLED-160G2, 4D Systems, Minchinbury, Australia) with the programmatically changeable spectrum was applied. Utilizing the chosen light source, a precise and selective illumination of the lab-chip could be achieved, maintaining uniform optical power in the area of interest. Dedicated software also allowed for the luminance regulation in the range of 70–100 Cd/m^2^.

An optical detection system on the platform (CCD camera, model: DLT-Cam PRO, Delta Optical, Warsaw, Poland) ensured constant observation of cells. On the basis of the acquired sample data, smart image processing could be initialized utilizing authorial software [31,32]. The implemented algorithms (*edge* and *blob detection*, *cell tracking*) provided cell detection and tracking at the single cell level, resulting in a complete characterization of the cultures in the context of population number, as well as cells’ size, shape, eccentricity, mobility, movement trajectory, and so on.

### 2.2. Cultured Microobjects

As mentioned earlier, different types of micro-objects have been studied, demanding specific and diverse culturing conditions. In the case of freshwater microorganisms, experiments encompassing both long-term culturing as well as taxis investigation were performed. As the development of *Euglena gracilis*, *Euglena viridis*, and *Lepadella patella*, in view of the predator–prey phenomenon, but also chemotaxis of *E. gracilis*, has been recently described in another paper [29]. In this article, the major attention was focused on the phototaxis surveys of *E. gracilis* (Blades Biological Ltd., Edenbridge, UK) using OLED irradiation.

Ovarian cancer cell line (SKOV-3) (American Type Culture Collection, Manassas, Virginia, USA) was cultured on the platform in the presence of constant medium flow (CO_2_-Independent Medium, Thermo Fisher Scientific, Waltham, MA, USA; supplemented with 10% FBS, 20 U penicillin, and 20 μg streptomycin/mL; Sigma-Aldrich, St. Louis, MO, USA) with the flow rate equaling 15 μL/min. The temperature of the module was set to 37 °C, according to the macroscale culturing standards [33].

In the case of porcine oocytes, the process of maturation (in vitro maturation, IVM) of these objects was performed on the LOC platform equipped with similar components as for cancer cell cultures, additionally including the source of gas mixtures. All of the experiments were compared to reference Petri dish cultures grown in 39 °C, 5% of humidity in CO_2_ incubator (model: HERAcell 150, Thermo Fisher Scientific, Waltham, MA, USA) [34].

Only preliminary research on mushroom representatives (*Cladosporium macrocarpum*) was performed on the LOC platform, but indicated high lab-on-chip material and technological biocompatibility.

## 3. Results

### 3.1. Euglena Gracilis Investigation

**Figure 5:**
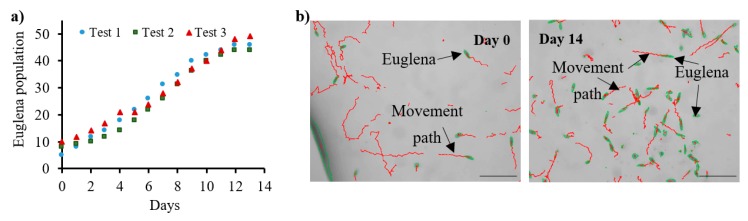
Culturing of *E. gracilis* on the LOC platform: (**a**) graph indicating exponential growth of these creatures, (**b**) microscope images of euglena colonies with software-enhanced detection of single cells and their movement paths. Micro-organisms were acquired from the macroscale culture and introduced into the LOC utilizing laboratory pipette (volume—1 μL) and covered by parafilm. The culturing process was performed in the middle microchamber under the daylight. Software processing was applied daily–fresh paths were generated each time. Scale bar—100 μm.

In view of the photosynthesis-based capabilities of *E. gracilis*, a 14-day culture of this micro-object was achieved by the constant delivery of CO_2_ (flow rate: 0.3 μL/min) to the chip microchambers (Figure 5). The exponential development of these creatures, reaching a plateau at the 12th day of the test, was consistent with the literature data [35,36]. In addition, appropriate, straightforward swimming character of *E. gracilis* during all the culturing time was observed (Figure 5).

**Figure 6:**
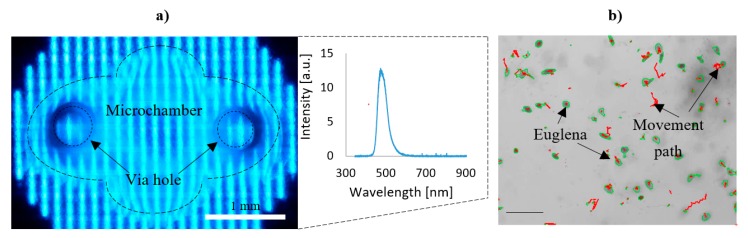
Study on *E. gracilis* phototaxis on the LOC platform: (**a**) view of the OLED illuminated microchamber with spectrum characteristics (blue color, 470 nm), (**b**) microscopic image of *E. gracilis* response to this illumination—visible rotational movements detected by the software. Luminance was set to 100 Cd/m^2^ for each test. Scale bar—100 μm.

Another experiment that was conducted on the LOC platform with a participation of *E. gracilis* referred to examination of photophobic responses of this microorganism [37,38]. Similar to the previous test, the *E. gracilis* sample was introduced into the chip chambers (1 and 2) and irradiated using the OLED matrix. Microchamber 1—by red color solely (favorable, reference wavelength of 615 nm), while microchamber 2—alternately by blue/red illumination (470/615 nm) for 5 min at each color. As a result (Table 2), in the case of microchamber 1, nearly equal mobility of euglenas was noticed, referring to typical behaviorism of these micro-objects. On the contrary, a different response of euglena was notified during the blue/red light switch on/off in microchamber 2. By applying the blue irradiation, a notable decrease in mobility occurred, revealing additional undesired, rotational movements of *E. gracilis* (Figure 6).

### 3.2. Ovarian Cancer Cells Development (Cell Line SKOV-3)

**Figure 7:**
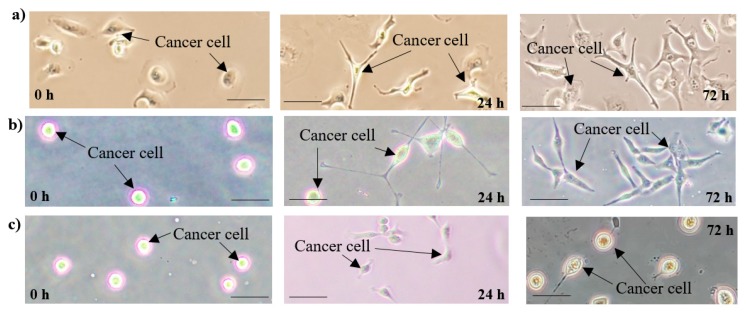
Microscope images of cancer cell cultures developed: (**a**) on a Petri dish in the incubator; (**b**) in the lab-on-chip device placed in the incubator (no medium flow), (**c**) utilizing the LOC platform (constant medium flow)—visible change in cells morphology. Scale bar—20 μm.

In this study, cultivation of the popular ovarian cancer cell line type SKOV-3 was performed on the LOC platform with the simultaneously run reference cultures utilizing standard incubator conditions. As the result of the experiments, an interesting change in cells’ morphology was observed after 24 and 72 h, probably a consequence of the constant flow provided within the microchamber area (Figure 7c). On the contrary, cell cultures established on-chip, but in the incubator, revealed the potential development similar to control cells cultured on a Petri dish (Figure 7a,b).

### 3.3. In Vitro Maturation (IVM) of Porcine Oocytes

In the following experiments, the process of IVM with a view to the first class porcine oocytes was conducted utilizing the LOC platform. The oocytes development, similar to the cancer cell cultures, was compared to the cultures established on-chip and on a Petri dish in the incubator. The temperature of the process was equal to 39 °C and flow rate did not exceed 15 μL/min. An additional element of the platform that had to be applied was the gas source, containing a mixture of CO_2_ and air in the relation of 95%/5%. The IVM process required a change of culturing medium as well, proceeding 22 h after the start of the maturation.

**Figure 8:**
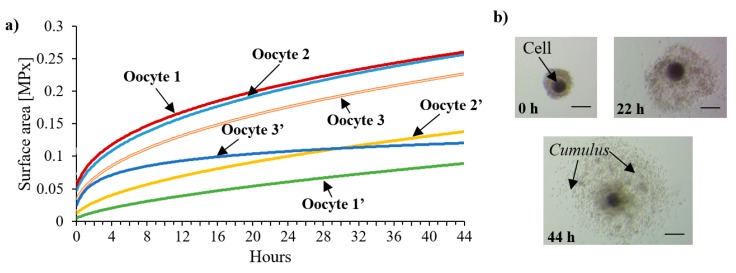
Development of porcine oocytes during the in vitro maturation (IVM) process performed on the LOC platform: (**a**) graph representing the expansion of oocytes’ size in two independent experiments, (**b**) microscope image indicating growth of *cumulus* cells. Scale bar—120 μm.

As shown in the graph (Figure 8), a notable growth of oocytes (considering especially the expansion of *cumulus* cells) could be noticed, being a qualitative evaluation of the well-fitted culturing conditions on the platform. Each of the matured oocyte increased in size by at least 100%, which was a similar result to the reference incubator cultures. After circa 34 h of IVM, oocytes’ expansion reached the plateau level, as indicated in the work of [39].

### 3.4. Culture of Cladosporium Macrocarpum

**Figure 9:**
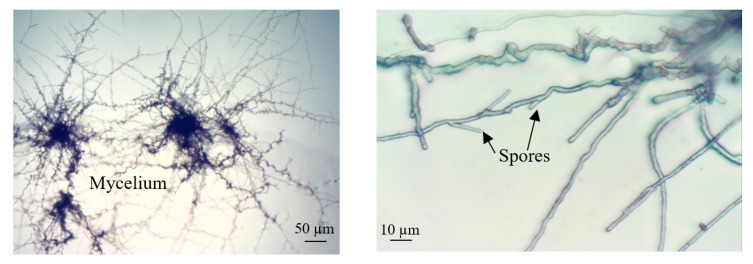
Microscope images of *C. macrocarpum* cultured in the all-glass LOC. Culturing conditions—ambient temperature, no lightning.

As mentioned earlier, solely lead-in studies on one of the most ubiquistic and cosmopolitan mushroom representatives—*Cladosporium macrocarpum*—were done on the LOC platform. As a result, appropriate physiological growth of these micro-objects could be observed using an all-glass lab-chip, which justifies its utility (Figure 9).

## 4. Discussion

In the paper, the universal microfluidic platform devoted to culturing and behavioral analysis of different biological objects has been presented. The platform uses all-glass LOCs and accompanying tools, ensuring the maintenance of cell cultures and their stimulations. Dedicated software provides qualitative and quantitative analysis of the biological samples, being a direct representation of cells’ behavioral change.

In reference to the results of the experiments conducted on the LOC platform, first of all, a long-term culturing of euglena species was achieved. In addition, phototaxis surveys were done, showing the visible photophobic responses of these creatures during blue light illumination (470 nm).

Culturing of ovarian cancer cells (SKOV-3) resulted in notable alternations in morphology, compared with the standard macroscale cultures of these objects. The cells lost their original fusiform shape and became more oval/ellipsoidal. It may reflect the features of epithelial morphology—typical for cells grown naturally in the human body [40]. However, these changes may also be the result of inadequately optimized culture parameters, for example, too high flow value of the culture medium that generates shear forces inducing oxidative stress and, ultimately, secondary cell shape changes. Further tests have to be done to unambiguously evaluate the potential development of cancer cell cultures on the LOC platform.

The experiments on the porcine oocytes IVM process confirmed the correct operation of all the platform components. According to the author’s best knowledge, the aforementioned IVM research, conducted outside a standard incubator, with simultaneous constant monitoring of the process parameters in real time, took place for the first time here. This solution certainly entangles within the latest trends on the construction of dynamic systems for the in vitro incubation of animals’ oocytes/embryos [41,42]. Such a field-deployable device could be widely utilized not only in most veterinary laboratories, but also in modern farms, increasing the potential development of the breeding animals.

Preliminary investigation on mushroom representatives *C. macrocarpum* showed adequate development of these objects, nevertheless, further studies must be done to formulate broader conclusions in this regard. Undoubtedly, studies concerning *fungi* currently constitute a highly important scientific branch, connected with the soil-on-chip trend as well [43].

## 5. Conclusions

In conclusion, a fully equipped and multipurpose microfludic platform applicable for diverse cell culturing experiments has been presented in this paper. The device allowed for long-term cultivation of the freshwater microorganisms, fungi, cancer cells, and animal oocytes, with a parallel driven real-time investigation of the biological samples’ development. The results of the experiments, in most of the cases, confirmed cells’ behavior as compliant with literature reports (only cancer cells culture exhibited interesting, notable morphological changes), which indicates the device operation to be appropriate.

It may be contended that the LOC platform presented here can be applied for different biomedical studies, and only small adaptable tasks have to be implemented to fit the specific researchers’ needs. The obtained results are a good base for the further platform development and widening its possible application horizons.

## Figures and Tables

**Table 1:** Summary of geometrical specification for each lab-on-chip (LOC).

Internal Structures Geometry						
	Overall Dimensions [mm]	Microchannel(s)		Microchamber(s)		Via Hole Diameter [mm]
		Depth	Width	Depth	Width	
**LOC 1**	76 × 26	5 μm	500 μm	100 μm	4 mm	1
**LOC 2**	35 × 17	400 μm	3.5 mm	-	-	2
**LOC 3**	76 × 26	150 μm	1 mm	150 μm	7 mm	1
**LOC 4**	50 × 25	80/400 μm	150/500 μm	-	-	1/1.5

**Table 2:** Comparison of *E. gracilis* mobility depending on the type of OLED irradiation in LOC microchambers.

	Microchamber 1		Microchamber 2	
Time [min]	Illumination [nm]	Mobility ^1^	Illumination [nm]	Mobility ^1^
0	Ambient	2.17	Ambient	2.17
5	615	↑2.23	470	↓1.54
10	615	↑2.33	615	↑2.07
15	615	↓2.22	470	↓1.65

^1^ Mobility is measured by the software as the sum of cells paths divided by no of cells in time in ROI. The value of mobility is a mean value taken from three tests (standard deviation did not exceed 5% here).
